# 

**DOI:** 10.1192/bjb.2024.21

**Published:** 2024-08

**Authors:** Muk Noong Cheng

**Affiliations:** An independent scholar in London, UK. Email: mnc8bma@gmail.com

**Figure:**
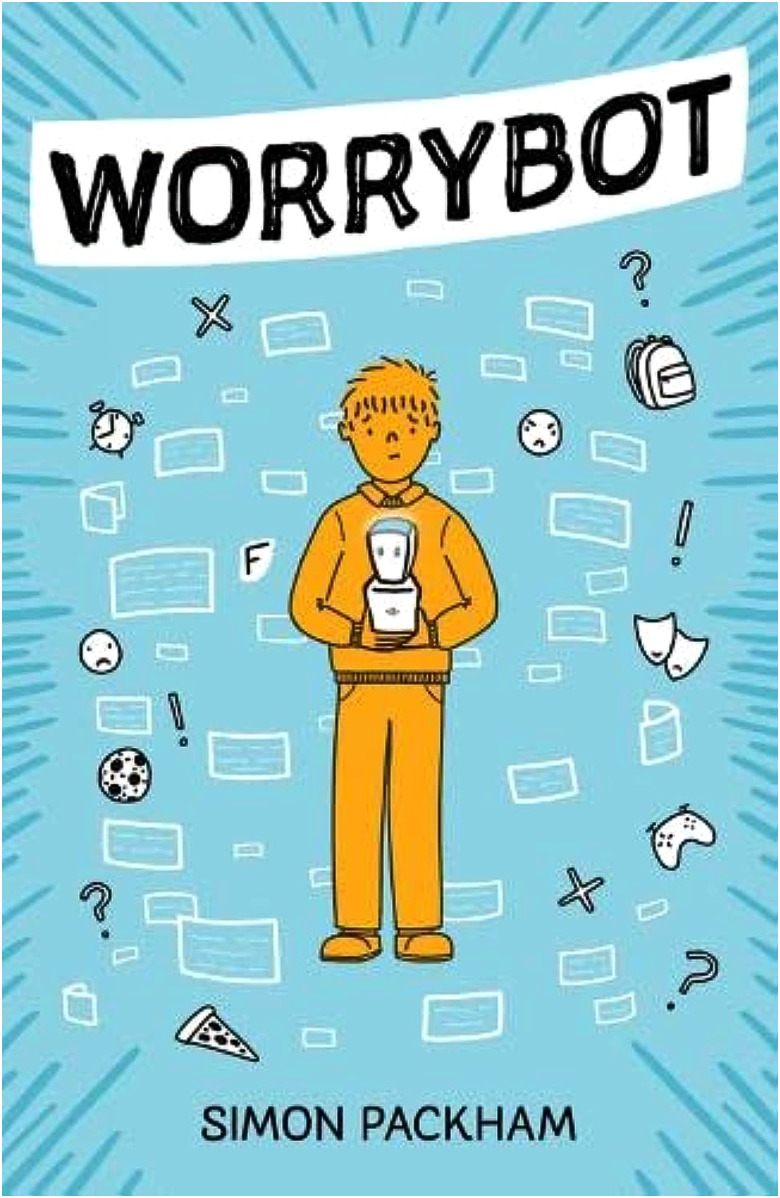

After a long and interesting career in child and adolescent psychiatry, I was very pleased to be asked to review *The Worrybot* by established children's author Simon Packham. Childhood anxiety disorders cause significant distress and often interfere with social, emotional and academic development. A minority of children with anxiety disorders will receive support from mental health practitioners, and teachers, who play an important part in early recognition and prevention of anxiety, are also overstretched.

However, anxiety itself is a universal human emotion, and we must be wary of over-medicalising it. One boy I know was worried during Covid that he had become obsessed with his ‘Happy Birthday handwashing’! Should we treat or should we understand more and find a better way out for the child than jumping to, say, the use of medication too early.

*Worrybot* is written from the perspective of 11-year-old Josh, who is terrified that his anxiety will resurface when he and his family move house and he changes school. The ‘Worrybot’ is a box where Josh can post his worries. At his new school, Josh is befriended by Charlie, a pupil who uses a learning robot to communicate with the rest of Year 6.

*Worrybot* is well written, with humour and plot twists designed to keep the young reader interested until the end. The central characters, Josh and Charlie, both experience significant anxiety symptoms, and the book tackles this issue in the context of bullying, friendships, family relationships and even bereavement in an elderly neighbour, in a sensitive and age-appropriate way.

The target audience is children aged 9 to 11 years, but I think teachers and parents could also benefit from reading it, and I found it as interesting (and rather more enjoyable) than many academic tomes covering the same topic. There are some real gems: early in the book we discover that other ‘patients’ have been posting their own troubles into the Worrybot – first Josh's mum (‘my performance management appraisal meeting’), then his dad (‘never getting another acting job’). There is also a very skilful description of the judicious use of a white lie. On Josh's first new school day, dad had no choice but to lie to rescue Josh from his newfound ‘friend’. The next day, dad forgot his lie, but luckily Josh's little sister remembered and lied herself to rescue her dad from the new friend's mum. They were taught early on that sometimes it's kinder not to tell the truth.

Simon Packham includes some strategies and links at the end of his book for overcoming anxiety. However, the main therapeutic effect is contained within the narrative. Children learn through play and stories, and my view is that this would be an ideal book to read individually or as a class group.

With the post-pandemic rise in ‘emotionally based school avoidance’, there surely is a place for a children's book which shows real understanding of the experience of anxiety, and also a way to live with it and still enjoy life.

I tip my hat to the *BJPsych Bulletin*'s decision to include a review of this book, as it has been my increasing realisation that there is a tendency in psychiatry to over-medicalise many childhood conditions, and, as the author demonstrates, there is scope to support children in finding their own solutions. One of the best lines in the book was ‘maybe taking advice from a school-refuser wasn't such a bad idea’. This links well with an ancient Chinese saying ‘a long sufferer will turn into a good doctor’.

As Picasso said, ‘I spent a lifetime trying to draw like a child’. Perhaps we should try to think like one.

